# Comparison of Horizontal *bla_CTX-M_* Gene Transfer via Conjugation among Extended Spectrum β-Lactamases Producing *Escherichia coli* Isolates from Patients with Urinary Tract Infection, Their Animals, and Environment

**DOI:** 10.33696/genetics.2.011

**Published:** 2023

**Authors:** Adam A. Mwakyoma, Benson R. Kidenya, Caroline A. Minja, Martha F. Mushi, Alison Sandeman, Wilber Sabiti, Mathew T. G Holden, Stephen E. Mshana

**Affiliations:** 1 Department of Bochemistry and Molecular Biology, Catholic University of Health and Allied Sciences, P. O. Box 1464, Bugando, Mwanza, Tanzania; 2 Department of Clinical Microbiology, Kilimanjaro Christian Medical Centre, P. O. Box 3010 Moshi, Kilimanjaro, Tanzania; 3 Department of Microbiology and Immunology, Catholic University of Health and Allied Sciences, P. O. Box 1464, Bugando, Mwanza, Tanzania; 4 School of Medicine, University of St Andrews, St Andrews, UK

**Keywords:** Horizontal gene transfer, Conjugation, ESBL –producing *E*. *coli*, *bla_CTX-M_* gene

## Abstract

**Background:**

The dissemination of the extended spectrum β-lactamases (ESBL) producing *E*. *coli* poses a significant public health problem. Understanding the efficiency and frequency of horizontal gene transfer via conjugation of ESBL producing *E*. *coli* is imperative towards devising prevention and control measures. This study compared the frequencies and efficiencies of horizontal *bla_CTX-M_* gene transfer via conjugation among *Escherichia coli* isolates from urine and gastrointestinal tract (GIT) of patients with urinary tract infection (UTI), their animals and environment.

**Methods:**

Horizontal *bla_CTX-M_* gene transfer via conjugation by a broth mating experiment was performed using 50 confirmed ESBL producing *E*. *coli* isolates as donors and *Escherichia coli* J53 (F^−^, *met*, *pro*, Az^r^), as the recipient. The transconjugants were detected and their frequencies and efficiencies of conjugation were measured and compared between ESBL producing *E*. *coli* isolates multi-sourced from urine, GIT, animals and environment. Antimicrobial susceptibility testing of all resulting transconjugants was performed. DNA was extracted from all transconjugants to confirm the presence and the acquisition of *bla_CTX-M_* gene.

**Results:**

Out of 50 ESBL producing *E*. *coli* isolates harboring *bla_CTX-M_* gene, 37 (74.0%) successfully exercised horizontal gene transfer through conjugation. All transconjugants were confirmed phenotypically and genotypically by PCR. Of note, all of the isolates from environment 100.0% (7/7) performed conjugation, exhibiting the highest transfer efficiency, followed by isolates from urine and animals, with the conjugation transfer efficiency of 77.8% (14/18) and 76.1% (10/13), respectively. The isolates from the environment conjugated with a significant more efficiency than those from the GIT [Two-sample test of proportions; p-value = 0.0119]. The overall conjugation transfer frequencies ranged from 0.4 × 10^-14^ – 5.5 × 10^-11^ per donor cells with the highest median conjugation transfer frequency observed among isolates from animal (3.23 × 10^-12^ [IQR: 0.70 × 10^-12^ – 7.22 × 10^-12^]) followed by that of isolates from the environment (1.60 × 10^-12^ [IQR: 0.30 × 10^-12^ – 5.0 × 10^-12^]).

**Conclusion:**

ESBL producing *E. coli* from human, animals and environment exercises horizontal *bla_CTX-M_* gene transfer efficiently with the highest occurrence among isolates from the environment and animals. The antimicrobial resistance control and prevention strategies should be widened up to explore strategies to prevent horizontal AMR gene transfer.

## Introduction

World Health Organization (WHO) considers the extended spectrum β-lactamase producing *Enterobactericeae* including *E. coli* as a *critical priority* for antibiotic development [1] and these pathogens are regarded as a *serious threat* by the US Centers for Disease Control [2]. The dissemination of extended spectrum β-lactamase (ESBL) producing *E. coli* poses a significant problem since it is the commonest causative agent of urinary tract infection (UTI) and is described to be the highest ESBL producing strain with *bla_CTX-M_* gene acquired via horizontal gene transfer [3,4]. The emergence of ESBL producing *E. coli* limits treatment options for UTI [5]. Furthermore, it has been reported that *E. coli* obtains antimicrobial resistance faster than other microorganisms [6].

Reports show that the ESBL genes such as *bla_CTX-M_'*, *bla_TEM_* and *bla_SHV_* have disseminated worldwide [3], of which *bla_CTX-M_* has been reported to be mostly disseminated through horizontal gene transfer [3]. Several genetic mechanisms have been involved in the spread of antimicrobial resistance genes and virulence genes, but conjugation is thought to have the greatest influence [4]. The mobile genetic elements such as conjugative plasmid, intergrons, and transposon are involved in dissemination of the antimicrobial resistance genes as well as virulence genes [3]. However, as compared to transformation and transduction, conjugation has been reported to be the mostly associated horizontal gene transfer mechanism via conjugative plasmid as the main vehicle for dissemination of antimicrobial resistance and virulence genes in both hospital and community settings [3,4]. Furthermore, studies have reported the horizontal gene transfer via conjugation disseminating antimicrobial resistance genes as well as virulence genes in *enterobacteriaceae* to occur in the human gut [7], animals [8] and environments [9]. This facilitates the spread of ESBL-producing *E. coli* across various ecological niches all over the globe and necessitates the surveillance of antimicrobial resistance to human, animals and environments.

Understanding the frequency and efficiency of horizontal gene transfer via conjugation among ESBL producing *E. coli* is imperative towards generating a better understanding of the mechanisms of dissemination of antimicrobial resistance genes in bacteria, an insight that is useful towards devising effective monitoring and prevention strategies against the dissemination of antimicrobial resistance genes. There is a high prevalence of the ESBL producing *E. coli* causing UTI and among those colonizing the GIT of human, animals, and environments [3,4, 10–13], suggesting the possibility of horizontal gene transfer for dissemination of the *bla_CTX-M_* gene via conjugation within these bacterial populations. However, there is scarce information regarding the frequency and efficiency of conjugation exercised by ESBL producing *E. coli* from urine, gastrointestinal tract, animals, and environments of patients with urinary tract infection. Therefore, this study aimed to determine and compare the frequencies and efficiencies of horizontal *bla_CTX-M_* gene transfer via conjugation among *E. coli* isolates from urine and gastrointestinal tract (GIT) of patients with UTI, their animals, and environment.

## Isolates, Materials and Methods

### Study isolates

A total of 50 ESBL producing *E. coli* were used as donors for horizontal gene transfer via conjugation with plasmid carrying *bla_CTX-M_* gene to the *Escherichia coli* J53 (F^−^, *met*, *pro*, Az^r^), a mutant strain of *E. coli* and a kind gift from the Institute of Medical Microbiology, Giessen, Germany, was used as a recipient [14]. Of the 50 ESBL producing *E. coli* isolates, 18 isolates were from urine of patients with UTI, 12 isolates were from GIT of patients with UTI, 13 were from the animals kept by these patients, and 7 isolates were from environment of these patients. All the 50 ESBL-producing *E. coli* donors were confirmed phenotypically and genotypically by PCR to harbor *bla_CTX-M_* gene. All the donors were activated overnight in Brain Heart infusion broth at 37°C ready for use in conjugation by a broth mating experiment.

### Horizontal *bla_CTX-M_* gene transfer via conjugation experiment

Conjugation of *bla_CTX-M_* gene from donor to recipient was performed using broth mating experiment as previously described [15] with slight modification. Briefly, the recipient strain which lacks *bla_CTX-M_* gene was prepared by streaking *Escherichia coli* J53 in Muller Hinton Agar (Himedia, India) plates supplemented with 100 μg/mL NaN3 [Sodium Azide], while donor strains were selected on Muller Hinton agar plates supplemented with 2 μg/mL Cefotaxime. From these, fresh overnight donor and recipient strains were prepared by picking single colonies emulsified in 10 mL Brain Heart Infusion (BHI) broth using different eppendorf tubes and incubated for overnight at 37°C. After exactly 12 hours, equal volumes (500 μL each) of donor and recipient strains were immediately mixed in 1.5 mL eppendorf tubes previously labeled transconjugant (Tc) while 1000 μL of donor strain were added in fresh tubes of similar volume to be separately selected on Muller Hinton agar plates supplemented with 2 μg/ _m_L Cefotaxime and Muller Hinton agar plates supplemented with 100 μg/mL NaN3 as respective controls. All tubes were incubated at 37°C for 15 minutes, vortexed briefly, centrifuged at 12,000×g for 2 minutes and the pellet re-suspended in fresh 1000 μL Brain Heart infusion broth. Finally, 100 μL of 10^−1^ to 10^-4^ transconjugant cultures were double selected on Muller Hinton Agar plates supplemented with 100 μg/mL NaN3 and 2 μg/mL Cefotaxime. Conjugation frequency (transconjugants per donor cells) was reported as transconjugants per donor cells, with the denominator obtained from an initial volume of 1000 μL. Conjugation frequency was calculated for serially diluted transconjugant and donor cells, then reported as transconjugants per donor cells with an initial volume of 1000 μL donor cells. Conjugation efficiency was measured in percent (%) and was obtained by taking the proportion of donor isolates that managed to transfer the *bla_CTX-M_* gene to recipient cells divided by the total number of donor isolates tested.

### Confirmation of horizontal *bla_CTX-M_* gene transfer via conjugation

#### Antibiotic susceptibility testing

Susceptibility testing of all resulting transconjugants was performed by the disk diffusion method on Mueller Hinton agar as recommended by the Clinical and Laboratory Standard Institute [16]. Antibiotics tested were: Cefotaxime (CTX) [2 μg/mL], Ciprofloxacin (CIP) [5 μg], Gentamicin (CN) [30 μg], Trimethoprim-Sulfamethoxazole (SXT) [1.25/23.75 μg] and Tetracycline (TET) [30 μg] (Hi-media, India).

#### DNA extraction of transconjugants

The boil lysate technique was used to extract bacterial DNA from isolates and selected transconjugants as previously reported [17] with slight modifications. Briefly, two colonies of overnight grown bacteria were suspended in brain heart infusion broth and incubated for six hours. The mixture was vortexed and boiled at 100°C in water-bath for 15 minutes. Tubes were centrifuged at 12000 rpm for 10 minutes and the DNA resuspended in 10 μl of nuclease free water. The quality of DNA was assessed by gel electrophoresis using TAE buffer while quantity was measured by qubit (Thermo Scientific). A minimum of 50 ng/μl was used for PCR reaction. Approximately 100 μl of DNA was aliquoted for storage at -20°C for further PCR amplification for detection of *bla_CTX-M_* gene.

#### PCR amplification for detection of *bla_CTX-M_* gene among transconjugants

Following DNA extraction, Polymerase Chain Reaction (PCR) was performed on thermal cycler machine (BIO-RAD, T100™, Singapore) for all transconjugants samples to amplify *bla_CTX-M_* gene using specific primers (Table 1) as previously described [8]. Briefly, 2 μl of DNA samples were added into PCR tubes containing a readily reconstituted master-mix (New England Biolabs) to make a final volume of 25 μl reaction mixture. PCR conditions were: initial denaturation 95°C for 15 minutes, followed by 30 cycles of denaturation at 94°C for 30 seconds, annealing at 60°C for 40 seconds, and elongation at 72°C for 2 minutes. A final extension step followed at 72°C for 10 minutes. Finally, PCR products were detected in 1.5% agarose gel (Invitrogen from Fisher Scientific, UK), 90 Amp, 100V, for 45 minutes using TAE buffer and visualized under UV light.

### Quality control

*Klebsiella pneumoniae* ATCC 700603, *Escherichia coli* ATCC 35218 and a clinical isolate of non ESBL *Escherichia coli* were used as control strains. These control strains were used to check the performance of media, antibiotic discs, as well as PCR experiments for amplification and detection of ESBL alleles.

### Data management and statistical analysis

Data from isolates such as identification number, isolate name, source of isolation, susceptibility pattern, conjugative frequency, and conjugative efficiency were recorded in the log book and then entered into the computer using Microsoft excel. Data were imported to STATA software version 15 for analysis. Kruskal-Wallis equality-of-populations rank test was used to compare the conjugation frequencies of ESBL-producing *E. coli* donors to the recipient between various sources of these isolates. Two-sample test of proportions was performed to compare the conjugation efficiency (Proportion) between isolates from the GIT which is the habitat for commensals *E. coli* with those from urine, animals, and environment. In all analyses the significance level was set at a p-value less than 0.05.

### Ethical clearance

The ethical clearance for this work was granted by the Joint Catholic University of Health and Allied Science –Bugando Medical Center (CUHAS-BMC) Ethical Review Committee (CREC).

## Results

### Conjugation efficiency and frequency of the *bla_CTX-M_ gene*

**Figure 1:**
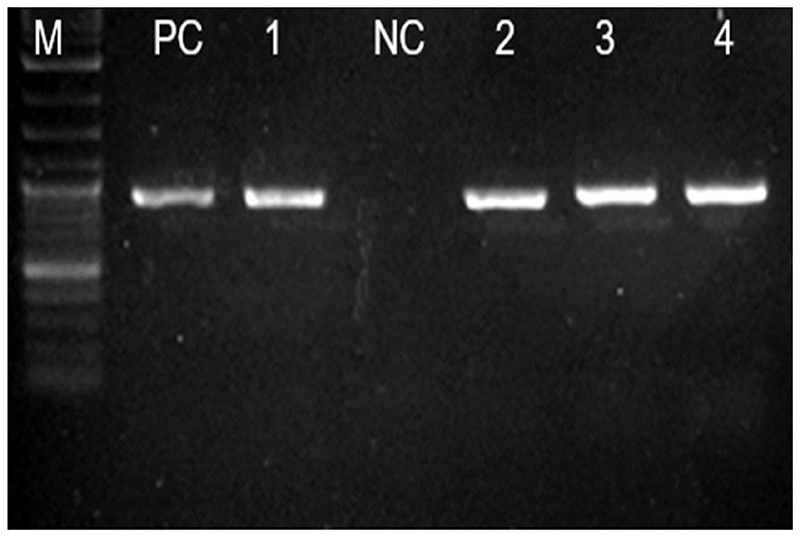
Gel image showing the *593 bla_CTX-M_* gene PCR product in 4transconjugants. Ladder: 100bp (New England BioLabs), Lane PC: Positive control *bla_CTX-M_* (*K. pneumoniae* ATCC 700603), Lane NC Negative control (Clinical isolate *E. coli* non ESBL), Lanes 1, 2, 3, and 4: *bla_CTX-M_* gene.

Out of the 50 ESBL producing *E. coli* isolates harboring *bla_CTX-M_* gene, 37 (74.0%) successfully transferred the gene via horizontal gene transfer through conjugation (Conjugation efficiency). All transconjugants were positive phenotypically and genotypically for *bla_CTX-M_* gene by showing a band of 593 bp on PCR (Figure 1). Of note, isolates from environment, performed conjugation with the highest transfer efficiency, as all of the isolates, 100.0% (7/7) successfully exercised conjugation transfer of *bla_CTX-M_* gene, followed by the isolates from urine and animals, with the conjugation transfer efficiency of 77.8% (14/18) and 76.1% (10/13) respectively. Of note, isolates from the environment conjugated with a significant more efficiency than those from the GIT [Two-sample test of proportions; p-value = 0.0119] (Table 2). The overall conjugation transfer frequencies ranged from 0.4 × 10^-14^ – 5.5 × 10^-11^ per donor cells. The highest median conjugation transfer frequency was observed among isolates from animal (3.23 × 10^-12^ [IQR: 0.70 × 10^-12^ – 7.22 × 10^-12^]) followed by that of isolates from the environment (1.60 × 10^-12^ [IQR: 0.30 × 10^-12^ – 5.0 × 10^-12^]). There was no statistically significant difference between the conjugation frequencies observed among the ESBL producing *E. coli* multi-sourced from urine, GIT, animals, and environment [Kruskal-Wallis equality-of-populations rank test; p-value = 0.8116] (Table 3). This emphasizes that ESBL producing *E. coli* performs horizontal *bla_CTX-M_* gene via conjugation at comparable frequencies.

### Antibiotic resistance phenotypes of donors from urine, GIT, animals, and environments and transconjugants

Of the 37 transconjugants detected, all (100.0%) expressed phenotypic drug resistance to Cefotaxime (CTX). Only 6 (16.2%) isolates expressed phenotypic resistance to Cefotaxime (CTX) alone yet their donor had also other antimicrobial resistance. Of note, there were 31 (83.8%) isolates that acquired and expressed antimicrobial resistance beyond Cefotaxime (CTX) from their donors. This includes resistance to ciprofloxacin (CIP), Gentamicin (CN), Trimethoprim-sulfamethoxazole (SXT), and Tetracycline (TET) in various combinations (Table 4).

## Discussion

The findings from this study reveal that ESBL producing *E. coli* from urine and gastrointestinal tract of patients with UTI, their animals, and environment exercise horizontal *bla_CTX-M_* gene transfer efficiently with the highest occurrence among isolates from the environment and animals. Furthermore, these isolates showed comparable conjugation transfer frequencies. This emphasizes that conjugation attributes to horizontal gene transfer for propagation of ESBL genes for drug resistance and underpins the intensification of antimicrobial surveillance from human to animals to environments.

The findings show that 74.0% of the ESBL producing *E. coli* from urine and gastrointestinal tract of patients with UTI, animals kept by these patients and their environment performed horizontal gene transfer via conjugation, with a 100% efficiency for the isolates from environment. This finding is similar to studies done by Minja et al., and Pérez-Etayo et al., in which the 88.1% and 100% respectively of the tested ESBL-producing *E. coli* strains, were able to perform an efficient gene transfer [15,19]. Furthermore, in Minja *et al.,* study all six ESBL producing *E. coli* isolates from the proper environment (soil) had a transfer efficiency of 100.0% similar to our finding. In our study the ESBL producing *E. coli* isolated from animals showed the highest conjugation transfer frequency. This highlights that environments and animals could be the most suitable ecological niche for ESBL-producing *E. coli* to mobilize and disseminate the antimicrobial resistance genes. Studies have shown that conjugation is one of the most important mechanisms for intra- and inter-species horizontal gene transfer, and it plays a significant role to accelerate the dispersal of antibiotic resistance genes [19–21]. Furthermore, ESBL genes have shown to be generally acquired by horizontal gene transfer [22] with some ESBL genes such as *bla_CTX-M_* gene mobilized from environmental bacteria [23]. For this reason, the fight against antibiotic resistance is a challenging and escalating problem liable to overwhelm our health systems. Several approaches are currently envisaged to combat the wide spread of antibiotic resistant pathogens particularly, by holding back the horizontal gene transfer. The continual search for specific conjugation inhibitors should be prioritized towards prevention of horizontal gene transfer of antimicrobial resistance as one of key target in the fight against the spread of antibiotic resistance genes [24].

When comparing the frequencies of conjugation for ESBL producing *E. coli* multi-sourced from urine, gastrointestinal tract of patients with UTI, their animals, and environment, we found there was no significant difference. This highlights that these ESBL producing *E. coli* perform horizontal *bla_CTX-M_* gene transfer via conjugation at comparable frequencies. This finding is also similar to the study done by Etayo *et al.,* and Minja *et al.,* which showed that the range of conjugation frequencies was nearly the same in all ESBL producing *E. coli* samples from various sources [15,18]. This habit fuels the acquisition of ESBL resistance genes across various ecological niches and gives the awaken call that the antimicrobial resistance surveillance, control and prevention strategies should be widen up to human, animals, and environment, as conjugation plays a major role in the spread of anti microbial resistance and the increasing occurrence of antibiotic resistance among pathogenic bacteria is considered a major problem for public health in recent decades [3,4,25–29].

In conclusion ESBL producing *E. coli* from urine and gastrointestinal tract of patients with UTI, their animals and environment exercise horizontal *blaC_TX-M_* gene transfer efficiently with the highest efficiency among isolates from the environment and highest frequency among isolates from animals. This emphasizes that conjugation appears to be a common phenomenon among ESBL producing *E. coli* and it attributes to horizontal gene transfer for propagation of ESBL genes for drug resistance. The antimicrobial resistance surveillance, control and prevention strategies should be widen up to human, animals and environment. Further studies are warranted in exploring the mobile genetic elements circulating in the environments and animals and the search for specific conjugation inhibitors should be prioritized towards prevention of horizontal gene transfer of antimicrobial resistance as one of key target in the fight against the spread of antibiotic resistance genes.

## Figures and Tables

**Table 1:** Primers targeting ESBL genes investigated in this study.

Gene targets	Primer name	Primer sequences	Amplicon
*bla_CTX-M_*	*bla_CTX-M_*_U_F	5'-ATGTGCAGYACCAGTAARGTKATGGC-3'	593 bp
	*bla_CTX-M_*_U_R	5'-TGGGTRAARTARGTSACCAGAAYCAGCGG-3'	

**Table 2:** ESBL producing *E. coli* conjugation transfer efficiency.

Source of ESBL – producing *E. coli*	Transconjugants Detected		p-value
	Yes	No	
	n (%)	n (%)	
Overall	37 (74.0)	13 (26.0)	-
GIT	6 (50.0)	6 (50.0)	Reference
Animal	10 (76.9)	3 (23.1)	0.0840
Urine	14 (77.8)	4 (22.2)	0.0868
Environment	7 (100.0)	0 (0.0)	0.0119

**Table 3:** ESBL producing *E. coli* conjugation transfer frequency.

Source of ESBL – producing *E. coli*	Median	Interquartile Range [IQR]	p-value
Overall	1.60 × 10^-12^	0.39 × 10^-12^ – 4.00 × 10^-12^	*-*
GIT	1.16 × 10^-12^	0.47 × 10^-12^ – 2.70 × 10^-12^	0.8116
Animal	3.23 × 10^-12^	0.70 × 10^-12^ – 7.22 × 10^-12^	
Urine	0.91 × 10^-12^	0.30 × 10^-12^ – 5.50 × 10^-12^	
Environment	1.60 × 10^-12^	0.30 × 10^-12^ – 5.00 × 10^-12^	

**Table 4:** Antibiotic resistance phenotypes of donors and transconjugants from urine, GIT, animals and environments.

SN	Sample ID	Sample Source	Conjugation frequency	Donors’ Resistance Phenotypes	Transconjugants’ Resistance Phenotypes
1	A1	ANIMAL	0.37 × 10^-12^	CTX + CIP + CN	CTX
2	A2	ANIMAL	0.39 × 10^-12^	CTX + CIP + CN	CTX + CIP
3	A3	ANIMAL	8.00 × 10^-12^	CTX + CIP + CN	CTX
4	A4	ANIMAL	0.07 × 10	CTX + CIP + CN	CTX + CIP + CN
5	A5	ANIMAL	0.40 × 10	CTX + CIP + CN	CTX + CIP + CN
6	A6	ANIMAL	3.86 × 10^-12^	CTX + CIP + CN	CTX + CIP + CN
7	A7	ANIMAL	2.60 × 10^-12^	CTX + CIP + CN	CTX + CIP + CN
8	A8	ANIMAL	0.86 × 10^-12^	CTX + CIP + CN	CTX
9	A9	ANIMAL	6.44 × 10^-12^	CTX + CIP + CN	CTX + CN
10	A10	ANIMAL	0.40 × 10	CTX + CIP + CN	CTX + CN
11	A11	ANIMAL	*-*	CTX + CIP + CN	*-*
12	A12	ANIMAL	*-*	CTX + CIP + CN	*-*
13	A13	ANIMAL	*-*	CTX + CIP + CN	*-*
14	G1	GIT	0.47 × 10^-12^	CTX + SXT + CIP + CN + TET	CTX + SXT + CIP + CN + TET
15	G2	GIT	1.71 × 10^-12^	CTX + CN	CTX + CN
16	G3	GIT	1.45 ×10^-11^	CTX + SXT + TET	CTX+TET
17	G4	GIT	0.60 × 10^-12^	CTX + SXT + CIP + CN + TET	CTX + SXT + CIP + CN + TET
18	G5	GIT	0.27 × 10	CTX + SXT + TET	CTX + SXT + TET
19	G6	GIT	0.20 × 10^-12^	CTX + SXT + CIP + CN	CTX + CN
20	G7	GIT	*-*	CTX + SXT + CIP + CN	*-*
21	G8	GIT	*-*	CTX + SXT + CIP	*-*
22	G9	GIT	*-*	CTX + SXT + CIP + CN	*-*
23	G10	GIT	*-*	CTX + SXT + TET	*-*
24	G11	GIT	*-*	CTX + CIP + CN	*-*
25	G12	GIT	*-*	CTX + SXT + CIP + CN	*-*
26	U1	URINE	0.72 × 10^-12^	CTX + SXT + CIP + CN + TET	CTX + SXT + CIP + CN + TET
27	U2	URINE	0.70 × 10^-11^	CTX + SXT + CIP + CN + TET	CTX
28	U3	URINE	0.01 × 10^-12^	CTX + SXT + CN + TET	CTX + SXT + CN + TET
29	U4	URINE	0.10 × 10^-12^	CTX + SXT + CN + TET + CIP	CTX + TET + CIP
30	U5	URINE	0.11 × 10^-11^	CTX + SXT + CN + TET + CIP	CTX + SXT + TET + CIP
31	U6	URINE	0.70 × 10^-11^	CTX + SXT + TET	CTX
32	U7	URINE	0.10 × 10^-12^	CTX + SXT + CIP + TET	CTX + CIP
33	U8	URINE	0.70 × 10^-12^	CTX + SXT + TET	CTX+TET
34	U9	URINE	0.20 × 10^-11^	CTX + SXT	CTX
35	U10	URINE	5.50 × 10^-11^	CTX + CIP + CN + TET	CTX + CN
36	U11	URINE	0.30 × 10^-12^	CTX + SXT + TET	CTX+TET
37	U12	URINE	0.30 × 10^-11^	CTX + SXT + TET	CTX+TET
38	U13	URINE	3.45 × 10^-13^	CTX + SXT + CIP + CN	CTX + CN
39	U14	URINE	0.50 × 10^-12^	CTX + SXT + CIP + TET	CTX + CIP + TET
40	U15	URINE	-	CTX + SXT + CIP + CN + TET	-
41	U16	URINE	-	CTX + SXT + CIP + CN	-
42	U17	URINE	-	CTX + SXT + CIP + TET	-
43	U18	URINE	-	CTX + SXT + CIP + TET	-
44	E1	ENVIRONMENT	0.40 × 10^-12^	CTX + CIP + CN	CTX + CIP + CN
45	E2	ENVIRONMENT	0.30 × 10^-12^	CTX + CIP + CN	CTX + CIP + CN
46	E3	ENVIRONMENT	0.50 × 10^-11^	CTX + CIP + CN	CTX + CIP + CN
47	E4	ENVIRONMENT	3.20 × 10^-12^	CTX + CIP + CN	CTX + CIP + CN
48	E5	ENVIRONMENT	1.23 × 10^-11^	CTX + CIP + CN	CTX + CIP + CN
49	E6	ENVIRONMENT	1.60 × 10^-12^	CTX + CIP + CN	CTX + CIP + CN
50	E7	ENVIRONMENT	0.40 × 10^-14^	CTX + CIP + CN	CTX + CIP + CN

SXT: Trimethoprim-sulfamethoxazole; CIP: Ciprofloxacin; TE: Tetracycline; CN: Gentamicin; CTX: Cefotaxime

## Data Availability

The raw data for our findings supporting the conclusion of this manuscript will be made available by the authors, without undue reservation, to any qualified researcher.
